# Editorial Department

**Published:** 1853-11

**Authors:** 


The second number of the Medical Reporter of Chester and Delaware counties, Pennsylvania, has reached us. It is a quarterly Journal of matters of professional interest, published under the direction of committees appointed by the societies of those counties. It embodies in the present number a topographical description of Chester county, and a tabular statement of deaths in both the counties of their jurisdiction. Aside from these, it has other interesting matter. Whether a journal so local can be supported may be doubted. That it *ought* to be well sustained, there is no question.

